# Supplementary material for: Evaluation of insecticide treated window curtains and water container covers for dengue vector control in a large-scale cluster-randomized trial in Venezuela
Source: PLoS Negl Trop Dis. 2022 Mar 4;16(3):e0010135. doi: 10.1371/journal.pntd.0010135 (PMC8926262; doi:10.1371/journal.pntd.0010135)
Supplement: S1 Table — a. Pupae/person Index summary. b. House Index Summary. c. Container index Summary. d. Breteau Index Summary. (DOCX) [file pntd.0010135.s001.docx]

**Supporting information**

**S1 Table**: Cluster-level means of each index in each arm at each survey.

**a: Pupae / person Index summary**

| **Arm** | **Follow-up** | | | | |
| --- | --- | --- | --- | --- | --- |
|  | **1** | **2** | **3** | **4** | **5** |
| **Control** | 0.083 | 0.182 | 0.204 | 0.013 | 0.145 |
| **ITC** | 0.134 | 0.195 | 0.127 | 0.087 | 0.330 |
| **ITJC** | 0.065 | 0.110 | 0.089 | 0.082 | 0.067 |
| **ITC+ITJC** | 0.047 | 0.003 | 0.072 | 0.047 | 0.033 |
| **External Control** | 0.221 | 0.336 | 1.115 | 0.113 | 0.907 |

**b: House Index Summary**

| **Arm** | **Follow-up** | | | | |
| --- | --- | --- | --- | --- | --- |
|  | **1** | **2** | **3** | **4** | **5** |
| **Control** | 7.17% | 11.67% | 6.26% | 3.92% | 6.80% |
| **ITC** | 7.87% | 6.90% | 3.04% | 4.58% | 14.24% |
| **ITJC** | 6.13% | 4.92% | 2.87% | 4.27% | 5.06% |
| **ITC+ITJC** | 6.78% | 0.39% | 3.16% | 5.95% | 4.78% |
| **External Control** | 11.44% | 13.33% | 21.05% | 3.51% | 26.79% |

**c: Container index Summary**

| **Arm** | **Follow-up** | | | | |
| --- | --- | --- | --- | --- | --- |
|  | **1** | **2** | **3** | **4** | **5** |
| **Control** | 4.24% | 7.20% | 4.44% | 3.56% | 4.79% |
| **ITC** | 6.23% | 4.86% | 1.65% | 2.33% | 10.02% |
| **ITJC** | 3.21% | 3.96% | 1.80% | 2.24% | 2.94% |
| **ITC+ITJC** | 3.29% | 0.18% | 2.08% | 1.76% | 3.27% |
| **External Control** | 6.15% | 8.04% | 16.49% | 2.19% | 24.66% |

**d: Breteau Index Summary**

| **Arm** | **Follow-up** | | | | |
| --- | --- | --- | --- | --- | --- |
|  | **1** | **2** | **3** | **4** | **5** |
| **Control** | 10.85 | 16.86 | 10.88 | 8.09 | 13.11 |
| **ITC** | 13.62 | 11.06 | 3.58 | 6.96 | 20.96 |
| **ITJC** | 8.50 | 15.20 | 4.33 | 6.45 | 8.53 |
| **ITC+ITJC** | 10.32 | 0.52 | 4.43 | 7.42 | 7.30 |
| **External Control** | 15.66 | 19.61 | 32.69 | 4.08 | 48.62 |
