# Supplementary material for: Evaluation of insecticide treated window curtains and water container covers for dengue vector control in a large-scale cluster-randomized trial in Venezuela
Source: PLoS Negl Trop Dis. 2022 Mar 4;16(3):e0010135. doi: 10.1371/journal.pntd.0010135 (PMC8926262; doi:10.1371/journal.pntd.0010135)
Supplement: Attachment — Submitted filename: PLoS DENCO Ven point by point response 03 NOV 2021.docx [file pntd.0010135.s002.docx]

Reviewer #1:

1.1 The methods are appropriate and the sample size calculations are fitting. The manuscript however refers to repeatedly to a spillover effect and uses and external control to partly assess that. This is all very well but it is difficult to assess without a clear description of the distance between clusters and whether or not buffer zones were included and received the intervention. An explicit description of the cluster design i.e. fried egg vs full gradient vs partial gradient would be very helpful in understanding the spillover. I would suggest including a schematic or a map with this information.

We have added a map of the study area (Figure 1), including the clusters by arm in the different towns, which should help clarify this point. The design did not use a fried egg or gradient design because the intention was to measure spillover effects within the clusters. The analytical methods for estimating spillover while taking account of cluster-randomised designs like this, were published only recently (Anaya-Izquierdo & Alexander, doi: 10.1111/biom.13316). Given the lengthy time since the Venezuelan study ended and the

complexity of the method, it was not applied in the current paper.

1.2 Data analysis: what is meant by "by definition, the randomized arms did not differ systematically at baseline" on page 7?

(Please see next point, 1.3)

1.3 I suggest including statistical testing of the entomological measurements at baseline presented in table 1.

We have followed an alternative approach which we believe to be more valid and informative, as explained in the following text, now added to the manuscript (Page 8, paragraph 1)

*“For the randomized arms, application of hypothesis tests would have been logically inconsistent because they would test whether any differences were due to chance, although the randomization ensured that they were due to chance. Instead, as a secondary analysis, we follow the recommendation of Altman & Doré and of Senn to include baseline values as a covariate in the regression model. More specifically, we used cluster-level quartiles of the baseline values as a categorical variable in each model.”*

1.4 Conclusions are generally supported by the results. Given the large difference between internal and external controls at baseline, I suggest de-emphasizing the community effect or better supporting this interpretation. Particularly in the abstract.

We agree on reducing the emphasis on the possibility of a community effect and the conclusions now read: *“ITMs can impact dengue vector populations in the long term, particularly when ITCs and ITJCs are deployed in combination.*

Reviewer #2:

2.1	The objectives of the study are clearly articulated with study design.

2.2	Study area
The authors could provide more general information about the breeding sites and productivity of Ae. aegypti, characteristics of water containers, house structure suitable for ITM deployment, as well as other socioeconomic characteristics and incidence of dengue to better describe and characterize the context of the study area.

We have added:

*Earlier studies in this area reported that large domestic water drums or barrels, 150—200litre in size, were producing, together with discarded tires, buckets and tanks, an estimated 70% of adult Ae. aegypti in the dry season and 80% in the wet season [24]. The majority of houses were concrete structures with relatively small windows which could be covered by ready-made curtains of fixed dimensions. Hence, the site was considered suitable for deployment of ITMs designed to target eclosion, oviposition and endophily in Ae. aegypti.*

2.3	Entomological surveys
Interventions were deployed in households and entomological surveys were conducted on households. Cluster Public spaces surrounding households also important places to be assessed, mosquitoes tend to be displaced from households to harbor other places, when principal breeding sites are intervened. Entomological surveys in public spaces were conducted? Do authors consider this information adds valuable information?

The clusters were almost exclusively residential and had been designed that way to avoid potential ‘contamination’ from non-residential sites. The importance of non-residential sites to *Aedes* production is well-documented, and at the time of this study we were particularly interested in the topic, having recently completed a study on vector output from a public cemetery, not far from this study site ^[1]^#. Hence, we tried to minimize ’contamination’ from public places by selecting geographic clusters that contained very few to zero non-residential sites.

2.4	Authors state: “BG Sentinel traps (Biogents AG, Germany) were originally deployed to monitor the adult mosquito populations in a small subset of houses in each study arm. However, due to persistent problems with the electrical supply and householder acceptability, the data collected was incomplete and their use was abandoned after the first follow-up survey”. Even though this method was abandoned I suggest mention the number of households that represent the small subset. Why when facing this barrier an alternative method for adult collection was not used?

There are notoriously few efficient options for collecting adult *Aedes* and vector biologists rarely agree on the sampling accuracy of traps like the BG, for epidemiological indices. Moreover, the project lacked the personnel and budget to conduct indoor aspirator collections, as the backpack sampling devices far exceeded our funds (this was some years before the affordable ‘Prokopack’ devices became available). This would have been our first option, but without it or some other method of collecting resting female mosquitoes it would not have been possible to sample adult mosquitoes in a meaningful way.

2.4	Serosurveys
The authors define that “Two serological surveys were undertaken to assess recent dengue infection. The first was conducted nine months after the ITMs were distributed (April 2007) and the second, 32 months after distribution (April 2009). All households with children under the age of 8 were invited to participate. Written informed consent was obtained from the head of household and a blood sample was obtained from one child under 8 years of age in each participating house. The blood sample was collected via finger capillary puncture and was blotted onto an individual piece of filter paper (Whatman)” Please clarify :

2.5	The first serological survey was conducted nine or eight months after ITMs distribution? Methods section mentions nine months while, results section 8 months.

Nine months is correct/ Results section has been corrected. (page 8, parag 2)

2.6	Why a baseline serological survey was not carried out?

We were trying to gauge protective efficacy during peak dengue transmission periods twice during the course of the study. Since IgM provides a measure of recent infection, we had hoped to detect any differences in recent infection between study arms during peak dengue transmission season.

2.7	What was the sample size calculated for this outcome?

As described in the Methods section, the study was powered on BI. However, with 1500 households targeted for inclusion in each treatment group and assuming a seroprevalence rate of 50% (realistic for high transmission areas), the study had ample power to detect differences of 5-8% between treatment groups.

2.8	“All samples were analyzed by dengue IgM capture ELISA at Mahidol University’s Centre for Vaccine Development in Bangkok, Thailand”.:
Suggest to write “enzyme linked immunosorbent assay” before ELISA

Done - bottom page 7

2.9	How results were classified: positive, negative or borderline?

2.10	What is the value of cutoff?

2.11	Which kit was used? Reagents? Reference?

Unfortunately, we have been unable to retrieve these details and, despite repeated efforts, we have been unable to locate or contact former colleagues in Bangkok who might have been able to help, but who are now retired.

2.12	Insecticide efficacy bioassays

Was Insecticide efficacy bioassays conducted with ITJC ? ITJC are exposed to other factors that may influence insecticide efficacy.

Bioassays weren’t conducted on ITJCs due to limitations in insectary capacity (limitations in space, mosquito output and personnel), so IT Curtains, which at the time, were considered the most likely to make an impact, were prioritized for the bioassays.

2.13	Insecticide susceptibility bioassays
Please clarify in method section how the results were categorized? Results section indicates “suspected resistant”

Added text (page 7):

*“WHO criteria were used to categorize bioassay results: 98-100% mortality = susceptible, 90-97% = suspected resistant, <90% = resistant.”*

2.14	Data analysis
Citation of R statistical package is missing. I suggest citation as follows: R Core Team (2020). R: A language and environment for statistical computing. R Foundation for Statistical Computing, Vienna, Austria. URL [https://www.R-project.org/](https://protect-eu.mimecast.com/s/jWQ0CKQDxU85VpETv5ZcA?domain=r-project.org)

Reference added

2.15	Randomization occurred among intervention and control clusters and arms did not differ systematically at baseline, but the study design is longitudinal and other intermediate factors may influence the effect.

Did the negative binomial regression model used considered possible intermediate variables? For example: climate, household variables or container variables that change over time, ITM conditions (exposition to sunlight, number of washes during the study period, damages, % of holes, coverage?

Please clarify if the effect of the intervention was adjusted for other intermediate variables that may be influencing the effect and it is not only due to the intervention.

In fact, we *had* already considered this query in text on page 9, parag 2, that reads:

“Since coverage was measured after randomization, including it in statistical analysis of the endpoints is likely to be misleading [33]. For example, high indices may result from low coverage of an intervention, or the reverse: poor performance of an intervention may cause trial participants to discontinue use. Some insight may be obtained from looking at temporal patterns of coverage and the indices, but this is complicated by the simultaneous variation in factors such as rainfall. Hence we report only descriptive analyses for coverage.“

2.16	Coverage
“Coverage of the interventions was defined per house as follows: in the ITC arm, as the proportion of houses with at least 1 ITC observed hanging at the time of the visit; in the ITJC arm, as the proportion appropriately using an ITJC at the time of the visit, with the denominator restricted to those with at least one drum; and in the combined ITC+ITJC arm, as the proportion using at least”
Time of the visit includes baseline?

There were no interventions in place at baseline.

2.17	What is the exact number of ITC and ITJC deployed per cluster? Per household?

This was based on the number of eligible containers and windows per house at any given time and as we replaced them when needed/requested by householders, varied between houses.  As such, the absolute numbers are not very informative (e.g., a house could have originally received 3 ITCs, then at the next visit they requested an additional one for a window they hadn’t previously wanted covered, and then at the next visit 2 of the ITCs were in poor condition but they only wanted 1 replaced, etc.). On average, houses allocated to the intervention arms received 4 ITCs and 1.5 ITJCs.

2.18	Was “poor performance of the intervention” measured?

For the ITCs, there’s the cone bioassay data.  For ITJCs, there was observational data that they were deteriorating as coverage declined, which was what led to the re-distribution at 20-months post-initial intervention.

2.19	Other variables of use, uptake and satisfaction of intervention measured? This will add information regarding recommendations about coverage. Some information is given in results section. O suggest to clarify the descriptive analysis carried out stating the variables presented in results section.

Outcomes associated with ITCs and their usage by communities, coverage and entomological impacts in this area, were explored in a related simultaneous study and have already been reported in a separate publication, cited in the manuscript:

Vanlerberghe et al. (2011) Evaluation of the Effectiveness of Insecticide Treated Materials for Household Level Dengue Vector Control. **PLoS NTDs** doi:10.1371/journal.pntd.0000994

2.20	Ethical approval
In some countries IRBs and local regulations require that young subjects how can read signed an assent form in addition to parental consent form. Is this a requirement in Venezuela ? If so please clarify.

At the time this study was performed, such approvals were not required by the ULA ethics committee, from whom all relevant approvals were obtained as state

2.21	Serological results
Interestingly if some Dengue incidence data is described during study periods

On p 13, the following text been added

*However, dengue transmission intensity tends to vary widely between years, and in the post-intervention survey, overall sero-prevalence was much lower than in the first survey. While dengue incidence appeared to be stable throughout the first year of the study, the numbers of reported cases in Trujillo State increased by up to 40% soon after introduction of the ITMs in April 2007, with levels sustained through the first ten epidemiological weeks of 2008 (Dirección Regional de Epidemiología y Estadística Vital, Trujillo-Venezuela, 2008).*

2.22	The serological surveys were carried 9 months or 8 months after intervention implementation?

9 months is correct; now corrected in Results

2.23	Range of age, mean age of among study arms and surveys are not reported.

We have been unable to obtains those detail at tis distance from the study.

2.24	Coverage
In results section: “The ITJCs experienced widespread physical deterioration, and many people had removed the ITCs to be washed and had not re-hung them. To address this, replacements for damaged or missing ITJCs were offered at the time of the entomological survey in April 2008 (20 months post- intervention)”
How many IT materials were replaced? By damage or missing per type ITC ITJC

These had been replaced *ad hoc* throughout the course of the study, but a larger re-distribution of ITJCs followed the 20-month survey due to significant loss arising from physical damage.

2.25	Conclusiones and limitations are clearly presented. However authors should clarify the following:
2nd paragraph last sentence reads as: “Despite the best efforts of the field team, coverage of the interventions never returned to the levels seen at the outset of the study, suggesting that the long-term use of ITMs by householders may be one of the greatest challenges to the success of their application”
Authors may add some further recommendations to assess this point. For example: should factors associated to use be assessed in depth. Have other studies assessed this?

We agree on the importance of this topic and the need to explore it in depth. Hence, these issues were considered in a separate earlier publication [37]:

Vanlerberghe et al. (2011) Evaluation of the Effectiveness of Insecticide Treated Materials for Household Level Dengue Vector Control. ***PLoS NTDs*** 5: e994 doi:10.1371/journal.pntd.0000994

2.25	“The second shortcoming is the inconclusive nature of the serological data collected to measure the impact of ITMs on dengue transmission. The dengue IgM ELISA was chosen to assess recent dengue infection in children under 8 years of age, under the assumptions that they would be less likely to have previously had dengue and would most likely be exposed to dengue in the home environment. However, dengue transmission intensity tends to vary widely between years, and in the post-intervention survey, overall sero-prevalence was much lower than in the first survey. Hence, given the low number of cases and insufficient power of the study, no comparison could be made between study arms.

How did dengue transmission vary during study periods (epidemic or endemic periods)?

This section of the discussion has been expanded as follows:

*However, dengue transmission intensity tends to vary widely between years, and in the post-intervention survey, overall sero-prevalence was much lower than in the first survey. While dengue incidence appeared to be stable throughout the first year of the study, the numbers of reported cases in Trujillo State increased by up to 40% soon after introduction of the ITMs in April 2007, with levels sustained through the first ten epidemiological weeks of 2008 (Dirección Regional de Epidemiología y Estadística Vital, Trujillo-Venezuela, 2008).*

2.26	Once reviewed the paper Evaluation of insecticide treated window curtains (ITM) and water container covers for dengue vector control in a large-scale cluster-randomized trial in Venezuela, I consider the work relevant and manuscript is worthy of publication. This study was well conducted and it is an important contribution to other evaluations of the effectiveness of ITM as an intervention to control mosquitoes. However, I have minimal suggestions and address several remarks to be clarified.

Abstract: This section is designed to highlight key points from major sections of the paper and to explain what the paper includes. Effective abstracts provide sufficient details to expedite classifying the paper as relevant (or not) to readers' clinical work or research interests, therefore
Additional information is needed to clarify the type of statistical model used.

Text has been added to the Abstract to state:

*“Negative binomial regression models were used to compare cluster-level values of these indices between the trial arms.”*

2.27	The full text describes as primary outcomes BI and PPI and as secondary outcomes ovitraps however the abstract reports that primary outcomes are: BI, PPI, CI, HI and ovitrap index).

Abstract changed to read: “*The Breteau and House indices were used as primary outcome measures and ovitrap indices as secondary.”*

2.28	I suggest to include the time frame when the study was conducted.

The text already reads “*The study began with household recruitment and a baseline survey in July 2006 and ran through until April 2009, when all follow-up surveys were completed.*

The dates are also shown in the figure and the CONSORT checklist indicates their inclusion.

2.29	Information about both insecticide efficacy and susceptibility is missing.

We are unsure of what might be missing here. The findings om ITM efficacy and vector population susceptibility are given in the final two sections of the Results. We have also updated the Methods section as requested.

2.30	Introduction
Second paragraph references are included as [4, 5, 6, 7] better (4-7). Please review writing guidelines,
Fourth paragraph [9, 10, 11, 12, 13] better 9-13

All have been amended.

3.1	In the Abstract/Conclusions the authors state: “ITMs can SUPPRESS dengue vector populations in the long term, EVEN WHEN COVERAGE IS SUBOPTIMAL, particularly when ITCs and ITJCs are deployed in combination.

I would recommend to the authors to eliminate SUPPRESS and to use another verb/word. Their intervention did not prove to suppress dengue vector populations in the long term.
Also, to change SUBOPTIMAL, unless it is clearly stated in the text what would be an “OPTIMAL” coverage.

See next point

3.2	Only a minor modification is suggested. In the Abstract/Conclusions the authors state: “ITMs can SUPPRESS dengue vector populations in the long term, EVEN WHEN COVERAGE IS SUBOPTIMAL, particularly when ITCs and ITJCs are deployed in combination. I would recommend to the authors to eliminate SUPPRESS and to use another verb/word. Their intervention did not prove to suppress dengue vector populations in the long term. Also, to change SUBOPTIMAL, unless it is clearly stated in the text what would be an “OPTIMAL” coverage.

Text has been edited and now reads simply:

*“ITMs can impact dengue vector populations in the long term, particularly when ITCs and ITJCs are depoyed in combination.”*

Figure Files:
While revising your submission, please upload your figure files to the Preflight Analysis and Conversion Engine (PACE) digital diagnostic tool, [https://pacev2.apexcovantage.com](https://protect-eu.mimecast.com/s/J-iyCN0JAiVm4ylIrmIE_?domain=pacev2.apexcovantage.com). PACE helps ensure that figures meet PLOS requirements. To use PACE, you must first register as a user. Then, login and navigate to the UPLOAD tab, where you will find detailed instructions on how to use the tool. If you encounter any issues or have any questions when using PACE, please email us at [figures@plos.org](mailto:figures@plos.org).

Data Requirements:
Please note that, as a condition of publication, PLOS' data policy requires that you make available all data used to draw the conclusions outlined in your manuscript. Data must be deposited in an appropriate repository, included within the body of the manuscript, or uploaded as supporting information. This includes all numerical values that were used to generate graphs, histograms etc.. For an example see here: [http://www.plosbiology.org/article/info:doi/10.1371/journal.pbio.1001908#s5](http://protect-eu.mimecast.com/s/b9rLCO8KBC5mWQRu5goZ6?domain=plosbiology.org).

Reproducibility:
To enhance the reproducibility of your results, we recommend that you deposit your laboratory protocols in [protocols.io](http://protect-eu.mimecast.com/s/E99KCPQLDUvEpVGfBt7hb?domain=protocols.io), where a protocol can be assigned its own identifier (DOI) such that it can be cited independently in the future. Additionally, PLOS ONE offers an option to publish peer-reviewed clinical study protocols. Read more information on sharing protocols at [https://plos.org/protocols?utm_medium=editorial-email&utm_source=authorletters&utm_campaign=protocols](https://protect-eu.mimecast.com/s/Ya0SCQ7METBj0ZACQ19nV?domain=plos.org)

References
Please review your reference list to ensure that it is complete and correct. If you have cited papers that have been retracted, please include the rationale for doing so in the manuscript text, or remove these references and replace them with relevant current references. Any changes to the reference list should be mentioned in the rebuttal letter that accompanies your revised manuscript. If you need to cite a retracted article, indicate the article's retracted status in the References list and also include a citation and full reference for the retraction notice.

1. # Abe et al. (2005) The Buen Pastor cemetery in Trujillo, Venezuela: measuring dengue vector output from a public area. ***Trop Med Int Health***, **10**; 597-603)
